# Beliefs about medication and their association with adherence in Chinese patients with non-dialysis chronic kidney disease stages 3–5

**DOI:** 10.1097/MD.0000000000028491

**Published:** 2022-01-14

**Authors:** He-He Bai, Xiao-Jing Nie, Xiao-Lin Chen, Ning-Jing Liang, Li-Rong Peng, Yan-Qin Yao

**Affiliations:** a Department of Pharmacy, Xi’ an Central Hospital, Xi’an, Shaanxi, China; b Department of Nephrology, Xi’ an Central Hospital, Xi’an, Shaanxi, China; c Department of Pharmacy, The Third Affiliated Hospital of Xi ’an Medical University, Xi’an, Shaanxi, China.

**Keywords:** beliefs about medication, medication adherence, non-dialysis CKD, psychometric properties

## Abstract

There is a scarcity of research into the impact of medication beliefs on adherence in patients with non-dialysis chronic kidney disease (CKD). This study is to determine the psychometric properties of the Chinese version of the Beliefs about Medicines Questionnaire (BMQ)-Specific among patients with non-dialysis CKD stages 3–5, and to assess the beliefs of CKD patients and their association with medication adherence.

A cross-sectional study was conducted in CKD patients who recruited at the nephrology clinics of Xi’an Central Hospital, Xi’an, Shaanxi, China. The original BMQ-Specific was translated into Chinese. The internal consistency and test–retest reliability of the Chinese version of the BMQ-Specific scale were assessed, while exploratory and confirmatory factor analyses were also applied to determine its reliability and validity. The Kruskal–Wallis test and multiple ordered logistic regression were performed to identify the relationship between beliefs about and adherence to medication among CKD patients.

This study recruited 248 patients. Cronbach's α values of the BMQ-Specific necessity and concern subscales were 0.826 and 0.820, respectively, with intraclass correlation coefficients of 0.784 and 0.732. Factor analysis showed that BMQ-Specific provided a good fit to the two-factor model. The adherence of patients was positively correlated with perceived necessity (*r* = 0.264, *P* < .001) and negatively correlated with concern (*r* = –0.294, *P* < .001). Medication adherence was significantly higher for the accepting group (high necessity and low concern scores) than for the ambivalent group (high necessity and concern scores; β = –0.880, 95% confidence interval [CI] = –1.475 to –0.285), skeptical group (low necessity and high concern scores; β = –2.620, 95% CI = –4.209 to –1.031) and indifferent group (low necessity and concern scores; β = –0.918, 95% CI = –1.724 to –0.112).

The Chinese version of BMQ-Specific exhibited satisfactory reliability and validity for use in patients with non-dialysis CKD stages 3–5 and has been demonstrated to be a reliable screening tool for clinicians to use to predict and identify the non-adherence behaviors of patients.

## 1 Introduction

Chronic kidney disease (CKD) is receiving increasing attention globally as an important public health problem. A cross-sectional survey showed that the prevalence of CKD was 10.8% in China, affecting an estimated 120 million people.^[1]^ The progression of CKD seriously impacts the health-related quality of life and imposes a financial burden. Regardless of whether or not they receive renal replacement therapy, patients with CKD often have multiple complications and co-morbidities, resulting in complex regimens involving multiple medications while increasing the likelihood of poor medication adherence.^[2]^ The term “medication adherence” refers to the extent to which a patient follows the medication regimen of his or her doctor or other healthcare provider.^[3]^ Medication adherence is an important determinant of whether treatment goals are attained in patients with CKD,^[4,5]^ and the reported prevalence of non-adherence to medication in patients with non-dialysis CKD has ranged from 17.4% to 53%.^[6,7]^ Medication non-adherence was shown to be associated with worse clinical outcomes such as increasing the risk of CKD progression and all-cause death in the Chronic Renal Insufficiency Cohort Study.^[8]^

Increasing the medication adherence requires an accurate understanding of the underlying causes of non-adherence. Non-adherence to medication is influenced by many factors, including those related to the medical condition, patient, therapy, health system, and socioeconomic domains.^[9]^ One factor whose influence on medication non-adherence in patients with non-dialysis CKD remains unclear is medication belief. Medication non-adherence is the result of reasonable decisions by the patient and the cognitive representation that is important to those decisions. The presence of beliefs about the necessity of taking medicine is associated with higher adherence.^[10]^ In contrast, the presence of concern about the adverse consequences of medication is more likely to lead to failure to comply with taking prescribed medication.^[11]^

The 8-item Morisky Medication Adherence Scale (MMAS-8)^[12]^ and the Beliefs about Medicines Questionnaire (BMQ)^[13]^ were developed for objectively measuring patient adherence to and beliefs about medication. MMAS-8 is frequently used for measuring medication adherence because of its simplicity, cost-effectiveness and operability. Some studies have applied MMAS-8 to Chinese patients, which can be well adapted to the psychological characteristics and meets the requirements of Chinese-speaking populations. Horne et al were the first to propose a measure based on a necessity-concern framework of BMQ to identify the beliefs of patients about medication.^[13]^ BMQ comprises 2 sections: BMQ-Specific and BMQ-General. BMQ-Specific is based on a necessity-concern framework, and is more closely related to adherence to medication than is BMQ-General (the more-general form of the scale).^[13]^ Beliefs about medication have been found to be a modifiable factor that are more predictive of adherence than are other social and clinical factors.^[14]^ Therefore, identifying the modifiable negative beliefs about medication might be an effective approach for clinicians to improve medication adherence in patients with non-dialysis CKD.

As a self-reported questionnaire, BMQ has been translated and verified in different cultural backgrounds and language environments, and its results are associated with other measures of adherence.^[15]^ The BMQ-Specific scale is widely used to evaluate the psychometric properties of medication beliefs in patients with chronic diseases,^[15,16]^ but it has not been applied to non-dialysis CKD patients who take multiple medicines. There is a general scarcity of research into the impact of medication beliefs on adherence in patients with non-dialysis CKD. Moreover, the scale has not been validated in Chinese patients with CKD. Therefore, the aims of the present study were (a) to determine the reliability and validity of the Chinese version of the BMQ-Specific scale in patients with non-dialysis CKD stages 3–5, and (b) to determine the beliefs of CKD patients and their association with medication adherence.

## 2 Methods

### 2.1 Study design and participants

This cross-sectional study was conducted in the nephrology clinic of a large general hospital (Xi’an Central Hospital, Xi’an, China) between March 1, 2018 and August 31, 2019. The study recruited 291 participants with non-dialysis CKD stages 3–5. Trained pharmacists and nurses of the hospital surveyed in- and outpatients and informed them about the purpose and content of the study. An informed-consent form was signed by all participants included in the study. The inclusion criteria were as follows:

1. older than 18 years,
2. diagnosed as CKD stages 3–5 (estimated glomerular filtration rate < 60 mL/min/1.73 m^2^) but not receiving dialysis, and
3. taking a single or multiple medications to control CKD stages 3–5 (including complications and co-morbidities) for at least 3 months.

Participants were excluded if they had a diagnosis of mental or psychosocial disease, cognitive impairment, cancer or failure of another important organ. This study was approved by the Institutional Review Board of Xi’an Central Hospital.

All of the participants were interviewed in a face-to-face manner by a trained pharmacist or nurse to complete the questionnaires. The questionnaires included the following information:

1. demographics and clinical characteristics (age, sex, marital status, education level, occupation, stage of CKD, duration of CKD stages 3–5, number of medications and experience of adverse drug reactions);
2. score on the BMQ-Specific scale;
3. score on the MMAS-8 scale; and
4. test–retest reliability of the BMQ-Specific scale, by randomly selecting 10% of the participants to complete the scale again after 2 to 4 weeks.

Finally, the adherence-related beliefs of the participants about medicines were assessed.

### 2.2 BMQ-specific

The BMQ-Specific scale was used to assess the beliefs of patients about medication. The original BMQ-Specific was independently translated to Chinese by 2 native Chinese speakers (a pharmacist and a medical doctor) who both were proficient in English and had academic and clinical experience. Both the translators and investigators discussed the translations and agreed on a final version, while focusing on producing a conceptually equivalent forward translation of the original questionnaire. An Irish native bilingual pharmacist who lived in China and spoke Chinese well performed a back translation of the BMQ-Specific scale (Chinese to English) while blinded to the content of the original BMQ-Specific scale. After completing the back translation, the original and back-translated versions were compared to identify potential discrepancies in meanings. The back-translated version only exhibited subtle semantic differences from the original questionnaire. The final version of BMQ-Specific was used in face-to-face interviews with 16 CKD patients.

The BMQ-Specific scale comprised 2 5-item subscales evaluating the beliefs of patients about the necessity of prescribed drugs for controlling chronic diseases and their concern about the potential adverse consequences.^[13]^ Each response item was scored on a 5-point Likert scale from 1 (“strongly disagree”) to 5 (“strongly agree”). The total scores on the necessity and concern subscales ranged from 5 to 25. The beliefs of patients about medicines represented by the 2 subscales were positively correlated with the scores. A subscale score of 15 were used as a cut-off for classifying into high and low necessity or concern.

The attitudes of patients toward medications could be divided into the following 4 groups to assess the relationship between beliefs about and adherence to medication:^[17]^

1. high necessity and high concern scores were classified into the ambivalent group,
2. high necessity and low concern scores were classified into the accepting group,
3. low necessity and high concern scores were classified into the sceptical group, and
4. low necessity and low concern scores were classified into the indifferent group.

A necessity–concern differential score (range –20 to 20) was calculated by subtracting the score on the concern subscale from that on the necessity subscale, due to the subscales of necessity and concern presenting different dimensions in the opposite direction. A higher differential score implies stronger beliefs in the necessity for medication than the concern about adverse consequences, and vice versa.^[18]^

### 2.3 MMAS-8

We used MMAS-8 to assess medication adherence in patients with non-dialysis CKD stages 3–5. Items 1 to 4, 6 and 7 on the scale are scored as 1 (“no”) or 0 (“yes”), item 5 is scored as 0 (“no”) or 1 (“yes”), and item 8 is measured on a 5-point Likert scale (1 = “never,” 0.75 = “once in a while,” 0.5 = “sometimes,” 0.25 = “usually” and 0 = “all the time”). The total scores ranged from 0 to 8, with a score of 8 indicating high adherence, scores of *<*8 and ≥6 indicating moderate adherence, and scores *<*6 indicating low adherence. Cronbach's α was found to be 0.556 for the internal consistency of Chinese version of the MMAS-8 by Yang et al^[19]^

### 2.4 Data analysis

The reliability of BMQ-Specific was assessed using Cronbach's α. A Cronbach's α of ≥0.70 is usually considered satisfactory. Intraclass correlation coefficients were used to evaluate test–retest reliability, with an ICC of >0.7 indicating a good reliability. Exploratory factor analysis (EFA) and confirmatory factor analysis (CFA) were conducted to assess the construct validity of BMQ-Specific and determine its suitability for Chinese speakers. EFA with geomin rotation was performed to examine whether the 10 items measured at least 1 of the constructs. Eigenvalues of >1 were used to decide the number of factors. CFA were conducted using Amos (version 24.0) software to evaluate whether the covariance among all item answers comprised 2 hypothesized factors or an alternate construct based on the EFA results. The goodness of fit was evaluated according to empirically supported criteria:^[20]^ the relative Chi-Squared (χ^2^/df) <3.0, the root-mean-square error of approximation <0.08, the standardized root mean squared residual (SRMR) <0.05, the comparative fit index > 0.90, the goodness of fit index and adjusted goodness of fit index > 0.90, the composite reliability (CR) > 0.7, the average of variance extracted (AVE) > 0.36.

Mean ± standard-deviation, median (range) and number (%) values were calculated using descriptive statistics for the demographic variables and clinical characteristics of the participants. Pearson's correlation coefficient was used to quantify the associations between MMAS-8 scores and scores on the BMQ-Specific subscales. The Kruskal–Wallis test was utilized to investigate the differences in medication adherence among the 4 attitudinal groups. Multiple ordered logistic regression was used to identify the factors that influence medication adherence. Independent variables for which *P* < .25 in the univariate analysis were included in a multivariate analysis model. We controlled for independent variables based on clinical experience: age, sex, marital status, education level, occupation, attitudes toward medication, experience of adverse drug reactions, stage of CKD, number of medications and duration of CKD stages 3–5. A *P* value of <.05 was considered statistically significant. All statistical analyses were conducted using the SPSS (version 24.0) program.

## 3 Results

### 3.1 Sample characteristics

Twenty four of the 299 CKD patients invited to participate in the study did not meet the inclusion criteria (19 because of having received dialysis treatment within the previous 3 months), 13 refused to participate in the study (10 because of unwillingness to provide basic information) and 8 withdrew from the study. In addition, 6 surveys were excluded due to being incomplete filled. Hence, 248 patients with non-dialysis CKD stages 3–5 were finally included in the study, corresponding to a dropout rate of 5.1%.

The included patients were aged 58.6 ± 17.4 years, with 54.4% being younger than 60 years. Additionally, 56.0% were male, 77.4% were married and 40.7% had retired. Moreover, 85.1% had received primary or secondary education, 19.0% reported that they had experienced an adverse drug reaction and 63.7% used more than 5 kinds of medications. The respondents had been diagnosed with CKD 4.8 ± 3.6 years previously. Their MMAS-8 score was 5.53 ± 1.95, and 15.7%, 30.6% and 53.6% of the respondents exhibited high, moderate and low adherence, respectively (Table 1).

**Table 1:** Demographics and clinical characteristics of the sample^∗^.

Characteristics	CKD Stage 3 60 > eGFR≥30 n = 102 (41.1%)	CKD Stage 4 30 > eGFR≥15 n = 86 (34.7%)	CKD Stage 5 eGFR < 15 n = 60 (24.2%)	Total n = 248
Age				
>60 yr	45 (44.1)	44 (51.2)	24 (40.0)	113 (45.6)
≤60 yr	57 (55.9)	42 (48.3)	36 (60.0)	135 (54.4)
Sex				
Male	59 (57.8)	47 (54.7)	33 (55.0)	139 (56.0)
Female	43 (42.2)	39 (45.3)	27 (45.0)	109 (44.0)
Marital status				
Single^†^	25 (24.5)	18 (20.9)	13 (21.7)	56 (22.6)
Married	77 (75.5)	68 (79.1)	47 (78.3)	192 (77.4)
Education level				
Higher education	15 (14.7)	13 (15.1)	9 (15.0)	37 (14.9)
Primary/secondary education	87 (85.3)	73 (84.9)	51 (85.0)	211 (85.1)
Occupation				
Farmer	26 (25.5)	17 (19.8)	8 (13.3)	51 (20.6)
Employed	26 (25.5)	18 (20.9)	18 (30.0)	62 (25.0)
Retired	37 (36.3)	38 (44.2)	26 (43.3)	101 (40.7)
Others^‡^	13 (12.7)	13 (15.1)	8 (13.3)	34 (13.7)
Duration of CKD				
≤5 yrs	70 (68.6)	60 (69.8)	44 (73.3)	174 (70.2)
5–10 yrs	20 (19.6)	18 (20.9)	9 (15.0)	47 (19.0)
>10 yrs	12 (11.8)	8 (9.3)	7 (11.7)	27 (10.9)
Number of medications				
≤5	35 (34.3)	31 (36.0)	24 (40.0)	90 (36.3)
>5	67 (65.7)	55 (64.0)	36 (60.0)	158 (63.7)
Experience of drug-related side effects				
Yes	26 (25.5)	14 (16.3)	7 (11.7)	47 (19.0)
No	76 (74.5)	72 (83.7)	53 (88.3)	201 (81.0)
MMAS-8 score, mean (SD)	5.86 (1.84)	5.46 (2.06)	5.05 (1.88)	5.53 (1.95)
High	22 (21.6)	13 (15.1)	4 (6.67)	39 (15.7)
Medium	31 (30.4)	30 (34.9)	15 (25.0)	76 (30.6)
Low	49 (40.0)	43 (50.0)	41 (68.3)	133 (53.6)

CKD = chronic kidney disease, eGFR = estimated glomerular filtration rate, MMAS = Morisky Medication Adherence Scale, SD = standard deviation.

∗ The data are reported as N (%) of patients unless otherwise indicated.

† Including divorced and widowed.

‡ Involving students, driver, merchant (trade), mechanic, guard.

### 3.2 BMQ-specific translation and measurements

**Figure 1:**
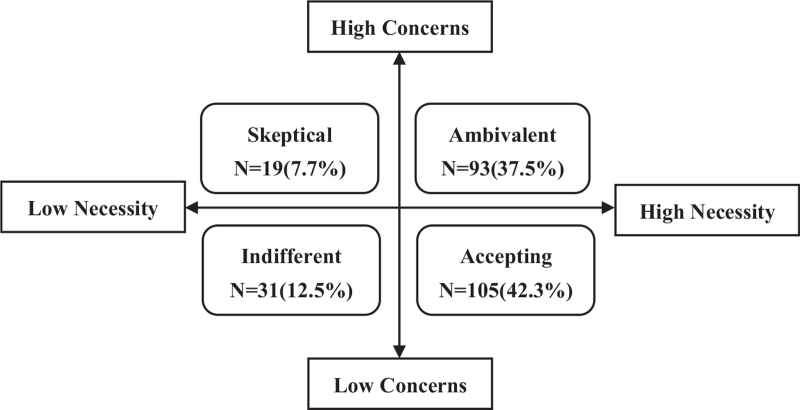
Distribution of the beliefs and attitudes of patients toward medication.

The results of the interviews of 16 CKD patients indicated that BMQ-Specific was understandable and readable for Chinese respondents. Patients with non-dialysis CKD stages 3–5 were more focused on the necessity of the prescribed medications (score of 17.46 ± 2.56) than being concerned about the potential adverse consequences (score of 14.87 ± 2.98). The necessity–concern differential score was 2.60 ± 3.97, ranging from –8 to 10. Among the 248 patients, 79.8% expressed the high necessity of medication, 45.2% had a high concern about medication (Fig. 1) and 20.6% had a negative value for the necessity–concern differential score.

### 3.3 Reliability

Cronbach's α for the necessity and concern subscales were 0.826 and 0.820, indicating satisfactory reliability of the Chinese version of BMQ-Specific. Cronbach's α decreased slightly when any single item was deleted. The test–retest reliability was calculated for 25 participants over a time interval of 18.32 ± 3.84 days. The intraclass correlation coefficients for the necessity and concern subscales were 0.784 and 0.732, respectively, which good test–retest reliability and reproducibility of the Chinese version of BMQ-Specific.

### 3.4 Construct validity

The *P* value in Bartlett test of sphericity was <.001 and the value in the Kaiser–Meyer–Olkin test was 0.803, which indicated that the Chinese version of BMQ-Specific was fit for factor analysis. Two factors (with eigenvalues >1) were extracted in the study, and they explained more than 50% of the data variance (Table 2). Absolute fit indices were used to test the hypothesis that the structure would fit the two-factor model. The relative Chi-Squared value (χ^2^/df) was 2.165. The goodness of fit index, adjusted goodness of fit index and comparative fit index were all larger than 0.90 (0.942, 0.907 and 0.951, respectively). The root-mean-square error of approximation was 0.069 and the SRMR was 0.045. These fit indices indicated that the model provided a good fit to the data.

**Table 2:** Factorial loading on the BMQ-specific^∗^.

	Factor 1	Factor 2
	Necessity	Concerns
Item4: Without my medicines I would be very ill	0.658	
Item3: My life would be impossible without my medicines	0.657	
Item1: My health, at present, depends on my medicines	0.642	
Item7: My health in the future will depends on my medicines	0.612	
Item10: My medicines protect me from becoming worse	0.58	
Item5: I sometimes worry about long-term effects of my medicines		0.647
Item2: Having to take medicines worries me		0.644
Item9: I sometimes worry about becoming too dependent on my medicines		0.626
Item8: My medicines disrupt my life		0.594
Item6: My medicines are a mystery to me		0.576
Eigenvalue	3.003	2.876
Explained variance (58.8%)	30.03%	28.76%

BMQ = beliefs about medicines questionnaire.

∗ Factor loadings < 0.5 are not presented in the table.

**Figure 2:**
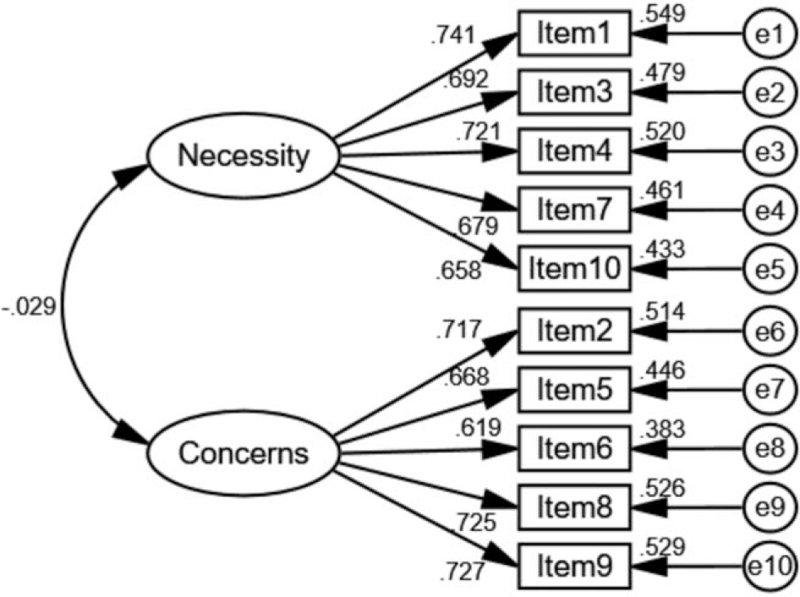
Confirmatory Factor Analysis of the BMQ-specific in non-dialysis CKD stages 3-5.

The results of the two-factor model are presented in Figure 2. All standardized factor loadings (>0.6) and standardized regression weights (>0.36) were acceptable in the model. The CR and AVE of the two-factor model were >0.7 and >0.36, respectively. The discriminant validity (DV) values among different dimensions were 0.699 and 0.693. These results show that the reliability and validity of the two-factor model were satisfactory in the BMQ-Specific scale.

### 3.5 Relationship between adherence and belief

Pearson's correlation analysis showed that the adherence score of patients was positively correlated with the necessity score (*r* = 0.264, *P* < .001) and the necessity–concern differential score (*r* = 0.391, *P* < .001), but negatively correlated with the concern score (*r* = –0.294, *P* < .001). Among the 4 attitudinal groups, 105 patients (42.3%) were accepting, 93 (37.5%) were ambivalent, 19 (7.7%) were skeptical and 31 (12.5%) were indifferent (Fig. 1). There were significant differences in adherence among the 4 attitudinal groups (χ^2^ = 38.519, *P* < .001). The MMAS-8 score was highest in the accepting group (6.35 ± 1.54) and lowest in the skeptical group (3.71 ± 1.94). Medication adherence were higher in the accepting group than in the ambivalent (*P* < .001), skeptical (*P* < .001) and indifferent (*P* = .007) groups (Table 3). In the multiple ordered logistic regression, patients with ambivalent (β = –0.880, 95% confidence interval [CI] = –1.475 to –0.285), skeptical (β = –2.620, 95% CI = –4.209 to –1.031) and indifferent (β = –0.918, 95% CI = –1.724 to –0.112) attitudes toward medication had significantly lower adherence compared with those who were accepting of medication, after adjusting for confounding factors such as experience of adverse drug reactions, number of medications, stage of CKD and duration of CKD (Table 4). In addition, patients who used more than 5 medications (β = –1.055, 95% CI = –1.688 to –0.421) and had experienced drug-related side effects (β = –0.744, 95% CI = –1.330 to –0.159) exhibited significantly lower adherence. Regarding the progression of CKD, CKD stage 4 (β = –0.101, 95% CI = –0.677 to 0.474) and CKD stage 5 (β = –0.836, 95% CI = –1.530 to –0.142) were associated with low adherence. A longer duration of CKD was associated with greater medication adherence, but this relationship was not statistically significant. In contrast, age, sex, marital status, education level and occupation were not associated with adherence.

**Table 3:** A Kruskal–Wallis single factor ANOVA test of the difference of medication adherence among attitudinal groups.

Attitudinal groups^∗^	MMAS-8 scores^†^	*P* value
Accepting	6.35 ± 1.54	<.001
Ambivalent	5.14 ± 1.91	
Skeptical	3.71 ± 1.94	
Indifferent	5.00 ± 1.95	
Accepting vs ambivalent		<.001
Accepting vs skeptical		<.001
Accepting vs indifferent		.007
Ambivalent vs skeptical		.058
Ambivalent vs indifferent		>.999
Skeptical vs indifferent		.204

MMAS = Morisky Medication Adherence Scale.

∗ Using the BMQ-specific scores with the level of necessity and concerns.

† Significant differences on adherence among the four attitudinal groups (χ^2^ = 38.519).

**Table 4:** Multiple ordered logistic regression of the factors associated with medication adherence.

	Crude^†^			Adjusted		
Variables	β coefficient	95% CI	*P* value	β coefficient	95% CI	*P* value
Attitudes toward medication^∗^						
Accepting	Reference			Reference		
Ambivalent	−1.162	−1,715, −0.608	<.001	−0.880	−1.475, −0.285	.004
Skeptical	−2.765	−4.290, −1.240	<.001	−2.620	−4.209, −1.031	.001
Indifferent	−0.962	−1.747, −0.177	.016	−0.918	−1.724, −0.112	.026
Number of medications						
≤5	Reference			Reference		
>5	−1.154	−1.756, −0.551	<.001	−1.055	−1.688, −0.421	.001
Experience of drug-related side effects						
No	Reference			Reference		
Yes	−1.035	−1.568, −0.502	<.001	−0.744	−1.330, −0.159	.013
Stage of CKD						
CKD 3	Reference			Reference		
CKD 4	−0.176	−0.719, 0.366	.524	−0.101	−0.677, 0.474	.730
CKD 5	−0.938	−1.591, −0.284	.005	−0.836	−1.530, −0.142	.018
Duration of CKD						
≤5 yrs	Reference			Reference		
5–10 yrs	0.383	−0.229, 0.994	.220	0.281	−0.375,0.937	.401
>10 yrs	0.526	−0.236,1.287	.176	0.504	−0.298,1.305	.218

CI = confidence interval, CKD = chronic kidney disease.

∗ Using the BMQ-specific scores with the level of necessity and concerns.

† A *P* value of <.25 in univariate analysis was included in the multivariate analysis.

## 4 Discussion

We assessed the reliability and validity of the Chinese version of BMQ-Specific in patients with non-dialysis CKD stages 3–5. This scale exhibited satisfactory reliability, and Cronbach's α values for the necessity and concern subscales were 0.826 and 0.820, respectively. These values are higher than the values obtained (0.55 and 0.73, respectively) when applying the original English version of the scale to renal dialysis inpatients.^[13]^ Several validation studies have been conducted for the BMQ-Specific scale in different cultures and languages.^[15,16]^ Cinar et al^[15]^ validated the Turkish version in patients with Behçet's disease, and Cronbach's α values for the necessity and concern subscales were 0.81 and 0.67, respectively. Tan et al^[16]^ verified the Malaysian version in hypertension patients, with Cronbach's α values for the necessity and concern subscales of 0.759 and 0.762, respectively. These values are similar to those that we obtained, which indicates that the Chinese version of BMQ-Specific has potential as a tool to help clinicians to assess the beliefs of non-dialysis CKD patients about medication.

EFA showed that the factorial structure of the Chinese version of BMQ-Specific was the same as that of the original English version and that each item loaded onto its expected factor. CFA also verified the hypothesis that the structure of the scale fit the two-factor model. All standardized regression weights squared were >0.4 except for item 6, which had a factor loading of slightly lower than 0.4. This lower loading may have been due to item 6 being phrased as “My medicines are a mystery to me,” whose multiple meanings in Chinese could have made it difficult for patients to answer. It has been reported that replacing the word “mystery” in the Danish and Swedish translated versions of the scale with “riddle” made the statement clearer.^[21]^ However, we did not modify the word “mystery” in the Chinese version in order to preserve the original meaning of the BMQ-Specific scale. The CR, AVE and DV of each subscale were acceptable, indicating that the Chinese version of BMQ-Specific had good convergent validity and DV in patients with non-dialysis CKD stages 3–5. The original BMQ scale has shown similarly satisfactory results in construct validity analyses among patients with various diseases, such as asthma, diabetes, kidney disease, heart disease and psychosis.^[13]^ It is therefore speculated that the versatility of the BMQ-Specific scale allows it to be applied to Chinese patients with other diseases.

This study has demonstrated the utility of the necessity- and concern-related framework in explaining medication non-adherence. High adherence among patients with non-dialysis CKD was associated with high necessity and low concern toward medication. Our results are consistent with Horne et al^[13]^ finding that the beliefs of patients can indeed be a powerful predictor of medication adherence. We discovered that the subjects in the 4 attitudinal groups exhibited significant differences in adherence, being highest in the accepting group (high necessity and low concern) and lowest in the skeptical group (high necessity and high concern). Patients with a higher level of health awareness for CKD are more likely to consider medication treatment necessary, which may have a positive impact on their adherence to medication.^[22]^ However, non-adherence is directly related to patients having excessive concerns about medication addiction, dependence, side effects and long-term toxicity.^[23]^ In our study, drug-related side effects played a particularly important role in non-adherence. Patients who experience side effects will often stop taking a medication, reduce the prescribed dose or apply complementary alternative medicines, which lead to lower adherence. Informing patients about known drug side effects and the countermeasures for dealing with them may be helpful for improving their beliefs about long-term medication treatment.

In addition, the number of medicines is always a determinant of medication adherence in patients with chronic diseases.^[24]^ Because CKD patients often have various co-morbidities (e.g., hypertension, diabetes and cardiovascular disease) and complications (e.g., anaemia, hyperkalaemia, osteodystrophy and acidosis), the use of multiple medicines can complicate their treatment regimens.^[25]^ A high burden of medicines will inevitably be associated with non-adherence behaviors, as demonstrated by our study finding that patients taking more than 5 medicines exhibited lower adherence. In terms of the severity of the disease, the adherence was lower among patients with CKD stage 5 than among those with CKD stages 3 and 4. Patients with CKD may suffer from an overwhelming psychologically burdened due to disease progression and the long-term effects of medicines. In many patients with CKD stage 5, medications do not provide significant symptom improvement and the necessity of taking medicines is often considered irrelevant, leading to negative beliefs about medication. A cohort study found that the non-adherence behaviors of patients were related to the progression of CKD,^[4]^ and it has also been found that worse kidney function is associated with a lower level of medication adherence.^[26]^ A particularly interesting finding in the present study was that a longer time since being diagnosed with CKD was associated with higher medication adherence. This result contradicts the finding of Naderi et al,^[27]^ and so further studies are needed to clarify this. Providing patients with a better understanding of CKD and a strong belief about their prescribed medicines are crucial to improving medication adherence.

This study was subject to several limitations. First, the study had a cross-sectional design, and so it could only explain adherence to medication at a certain time, whereas this can change over time. Second, although self-reporting is the most appropriate method for evaluating medication adherence, only using a self-reported questionnaire on medication adherence may lead to bias in the results. Third, the included patients were recruited at a medical institution, which may have led to overestimation of the adherence rate. Fourth, we only studied the specific beliefs about medicines in patients with non-dialysis CKD stages 3–5, and there was a lack of evidence for the influence of general beliefs on medication adherence. Finally, the study population might not represent the medication beliefs of all patients with chronic diseases. Therefore, future studies need to investigate patients with other diseases in order to confirm the relationship between the beliefs of patients and their adherence to medication.

## 5 Conclusions

The present study found strong evidence for the reliability and validity of the Chinese version of BMQ-Specific, making it suitable for use in patients with non-dialysis CKD stages 3–5. The beliefs of patients about medication have an important impact on their adherence, thus influencing whether they actively participate in their own CKD management. The Chinese version of the BMQ-specific has been demonstrated to be a reliable screening tool for clinicians to use to predict and identify the non-adherence behaviors of patients.

## Author contributions

**Conceptualization:** He-he Bai, Yan-Qin Yao.

**Data curation:** He-he Bai, Xiao-Jing Nie, Xiao-Lin Chen, Ning-Jing Liang.

**Investigation:** He-he Bai, Xiao-Lin Chen, Ning-Jing Liang.

**Methodology:** He-he Bai, Xiao-Jing Nie, Xiao-Lin Chen, Ning-Jing Liang.

**Validation:** He-he Bai, Li-Rong Peng.

**Writing – original draft:** Xiao-Jing Nie, Yan-Qin Yao.

**Writing – review & editing:** He-he Bai, Yan-Qin Yao.

## References

[1] Zhang L Wang F Wang L. Prevalence of chronic kidney disease in China: a cross-sectional survey. Lancet 2012;379:815–22. 22386035 10.1016/S0140-6736(12)60033-6

[2] Parker K Bull-Engelstad I Aasebø W. Medication regimen complexity and medication adherence in elderly patients with chronic kidney disease. Hemodialysis Int 2019;23:333–42. 10.1111/hdi.12739 30779285

[3] Morisky DE Green LW Levine DM. Concurrent and predictive validity of a self-reported measure of medication adherence. Medical Care 1986;24:67–74. 3945130 10.1097/00005650-198601000-00007

[4] Tangkiatkumjai M Walker DM Praditpornsilpa K Boardman H. Association between medication adherence and clinical outcomes in patients with chronic kidney disease: a prospective cohort study. Clin Exp Nephrol 2016;21:01–9. 10.1007/s10157-016-1312-6 27438073

[5] Tesfaye WH McKercher C Peterson GM. Medication adherence, burden and health-related quality of life in adults with predialysis chronic kidney disease: a prospective cohort study. Int J Environ Res Public Health 2020;17:371. 10.3390/ijerph17010371 PMC6981524 31935851

[6] Hsu KL Fink JC Ginsberg JS. Self-reported medication adherence and adverse patient safety events in CKD. Am J Kidney Dis 2015;66:621–9. 25979348 10.1053/j.ajkd.2015.03.026 PMC4586079

[7] Magacho EJC Ribeiro LC Chaoubah A Bastos MG. Adherence to drug therapy in kidney disease. **Braz** J Med Biol Res 2011;44:258–62. 21344138 10.1590/s0100-879x2011007500013

[8] Cedillo-Couvert EA Ricardo AC Chen J. Self-reported Medication Adherence and CKD Progression. Kidney Int Rep 2018;3:645–51. 29854972 10.1016/j.ekir.2018.01.007 PMC5976857

[9] Seng JJB Tan JY Yeam CT Htay H Foo WYM. Factors affecting medication adherence among pre-dialysis chronic kidney disease patients: a systematic review and meta-analysis of literature. Int Urol Nephrol 2020;52:903–16. 32236780 10.1007/s11255-020-02452-8

[10] Jamous RM Sweileh WM Taha EDA Zyoud SEH. Beliefs about medicines and self-reported adherence among patients with chronic illness: a study in palestine. J Family Med Prim Care 2014;3:224–9. 25374859 10.4103/2249-4863.141615 PMC4209677

[11] Carlos DLC Mariano M Trino B Jose DL. Skepticism and pharmacophobia toward medication may negatively impact adherence to psychiatric medications: a comparison among outpatient samples recruited in Spain, Argentina, and Venezuela. Patient Prefer Adherence 2018;12:301–10. 29503532 10.2147/PPA.S158443 PMC5824753

[12] Morisky DE Ang A Krousel-Wood M Ward HJ. Predictive validity of a medication adherence measure in an outpatient setting. J Clin Hypertens 2008;10:348–54. 10.1111/j.1751-7176.2008.07572.x PMC2562622 18453793

[13] Horne R Weinman J Hankins M. The beliefs about medicines questionnaire: The development and evaluation of a new method for assessing the cognitive representation of medication. Psychol Health 1999;14:01–24.

[14] Emilsson M Gustafsson PA Ohnstrom G Marteinsdottir I. Beliefs regarding medication and side effects influence treatment adherence in adolescents with attention deficit hyperactivity disorder. Eur Child Adolesc Psychiatry 2017;26:559–71. 27848023 10.1007/s00787-016-0919-1 PMC5394130

[15] Cinar M Cinar F Acikel C. Reliability and validity of the Turkish translation of the beliefs about medicines questionnaire (BMQ-T) in patients with Behçet's disease. Clin Exp Rheumatol 2016;34:S46–51. 27191774

[16] Tan CS Hassali MA Neoh CF Saleem F Horne R. Cultural adaptation and linguistic validation of the beliefs about medicines questionnaire in Malaysia. Value Health Reg Issues 2018;15:161–8. 29730249 10.1016/j.vhri.2017.12.010

[17] Park HY Seo SA Yoo H Lee K. Medication adherence and beliefs about medication in elderly patients living alone with chronic diseases. Patient Preference Adherence 2018;12:175–81. 29416319 10.2147/PPA.S151263 PMC5790098

[18] Horne R Chapman SC Parham R Freemantle N Forbes A Cooper V. Understanding patients’ adherence-related beliefs about medicines prescribed for long-term conditions: a meta-analytic review of the Necessity-Concerns Framework. PLoS One 2013;8:e80633. 24312488 10.1371/journal.pone.0080633 PMC3846635

[19] Yang A Wang B Zhu G. Validation of Chinese version of the Morisky Medication Adherence Scale in patients with epilepsy. Seizure 2014;23:295–9. 24484672 10.1016/j.seizure.2014.01.003

[20] Hu Lt Bentler PM. Cutoff criteria for fit indexes in covariance structure analysis: conventional criteria versus new alternatives. Struct Equ Model 1999;6:01–55.

[21] Granas AG Nørgaard LS Sporrong SK. Lost in translation?: Comparing three scandinavian translations of the beliefs about medicines questionnaire. Patient Educ Couns 2014;96:216–21. 24908591 10.1016/j.pec.2014.05.010

[22] Wong KK Velasquez A Powe NR Tuot DS. Association between health literacy and self-care behaviors among patients with chronic kidney disease. BMC Nephrology 2018;19:196. 30081951 10.1186/s12882-018-0988-0 PMC6091174

[23] Tangkiatkumjai M Kefale B Tadesse Y Alebachew M Engidawork E. Management practice, and adherence and its contributing factors among patients with chronic kidney disease at Tikur Anbessa Specialized Hospital: A hospital-based cross-sectional study. PLoS One 2018;13:e0200415. 30044830 10.1371/journal.pone.0200415 PMC6059431

[24] Burnier M Pruijm M Wuerzner G Santschi V. Drug adherence in chronic kidney diseases and dialysis. Nephrol Dial Transplant 2014;30:39–44. 24516224 10.1093/ndt/gfu015

[25] Parker K B-EI Aasebø W. Medication regimen complexity and medication adherence in elderly patients with chronic kidney disease. Hemodial Int 2019;23:333–42. 30779285 10.1111/hdi.12739

[26] Schmitt KE Edie CF Laflam P Simbartl LA Thakar CV. Adherence to antihypertensive agents and blood pressure control in chronic kidney disease. Am J Nephrol 2010;32:541–8. 21042012 10.1159/000321688

[27] Naderi SH Bestwick JP Wald DS. Adherence to drugs that prevent cardiovascular disease: meta-analysis on 376,162 patient. Am J Med 2012;125:882–7. 22748400 10.1016/j.amjmed.2011.12.013

